# A Greener, Quick and Comprehensive Extraction Approach for LC-MS of Multiple Mycotoxins

**DOI:** 10.3390/toxins9030091

**Published:** 2017-03-07

**Authors:** Andreas Breidbach

**Affiliations:** Andreas Breidbach, European Commission—Joint Research Centre—Directorate F—Health, Consumers, and Reference Materials, Retieseweg 111, B-2440 Geel, Belgium; Andreas.breidbach@ec.europa.eu; Tel.: +32-14-571-205

**Keywords:** mycotoxins, LC-MS, green analytical chemistry

## Abstract

In food/feed control, mycotoxin analysis is often still performed “one analyte at a time”. Here a method is presented which aims at making mycotoxin analysis environmentally friendlier through replacing acetonitrile by ethyl acetate and reducing chemical waste production by analyzing four mycotoxins together, forgoing sample extract clean-up, and minimizing solvent consumption. For this, 2 g of test material were suspended in 8 mL water and 16 mL ethyl acetate were added. Extraction was accelerated through sonication for 30 min and subsequent addition of 8 g sodium sulfate. After centrifugation, 500 µL supernatant were spiked with isotopologues, dried down, reconstituted in mobile phase, and measured with LC-MS. The method was validated in-house and through a collaborative study and the performance was fit-for-purpose. Repeatability relative standard deviation (RSDs) between 16% at low and 4% at higher contaminations were obtained. The reproducibility RSDs were mostly between 12% and 32%. The trueness of results for T-2 toxin and Zearalenone were not different from 100%, for Deoxynivalenol and HT-2 toxin they were larger than 89%. The extraction was also adapted to a quick screening of Aflatoxin B1 in maize by flow-injection–mass spectrometry. Semi-quantitative results were obtained through standard addition and scan-based ion ratio calculations. The method proved to be a viable greener and quicker alternative to existing methods.

## 1. Introduction

Mycotoxins are toxic secondary metabolites of certain moulds whose occurrence in food and feed is difficult to avoid. They present a potential risk to the health of consumers. Therefore, many countries have regulated the occurrence of mycotoxins [1,2]. A wealth of methods of analysis exists to enforce these regulations. Many of these methods rely on mixtures of acetonitrile (ACN)/water for extraction of the mycotoxins from the solid food/feed matrix [3]. To minimize sub-sampling uncertainties, test portion sizes of 25–50 g are not uncommon, which are then extracted with tens or hundreds of mL of extraction solvent [4]. In the end, only an aliquot of the extract is cleaned up and actually used for the determination. The rest is chemical waste needing proper disposal.

A compounding factor for the waste problem is the need to prepare the test material several times to determine several mycotoxins in the “classical” way. Classical approaches in mycotoxin analysis make use of immuno-affinity clean-up to obtain the necessary selectivity prior to separation/detection with HPLC with ultraviolet (UV) or fluorescence detector. These methods are mostly used for single or, at most, very few, very similar analytes.

“Green Analytical Chemistry” (GAC) is a rapidly developing trend that is rooted in the desire to make chemical analysis environmentally friendlier. Key principles are, amongst others, chemical waste reduction and the use of “safer” solvents [5]. One way of reducing the amount of chemical waste produced during food/feed analysis is certainly decreasing test portion size. With a smaller test portion, the volume of extraction solvent can be reduced while maintaining a high solvent/sample ratio beneficial for good extraction yields. This requires a higher effort in homogenizing the sample which is delivered to the laboratory to avoid sub-sampling uncertainties which might exceed the measurement uncertainty. Sample materials milled to particle sizes smaller than 500 µm and thoroughly mixed usually fulfil this requirement. Another approach would be to prepare aqueous slurries. For this, the test material and a certain amount of water are high-speed blended to create a well-mixed dispersion of very small particles. Such slurries display very little heterogeneity and thus minimize the sub-sampling uncertainty as shown by Spanjer et al. for 10 kg samples [6]. Preparing slurries of 10–50 g of test material and then using a test portion which is the slurry equivalent of 1 or 2 g of test material would be a compromise. The limited amount of unused slurry consisting of water and test material is easy to dispose of and the small aliquot of slurry which is extracted only requires a relatively small volume of organic solvent. Yet the sub-sample size is that of older, proven methods.

Increasing the number of analytes determined per analysis from the same test material preparation also reduces the generation of chemical waste. In mycotoxin analysis, modern generation LC-MS with high sensitivity and selectivity enable this [7] and the selectivity of LC-MS also allows the analyst to forgo sample extract clean-up, which eliminates another waste-producing step [8,9]. Another advantage of LC-MS is its preference for lower flow rates. While in the classical HPLC analysis, analytical columns of 4.6 mm i.d. at flow rates of 1 mL/min are used; LC-MS, with the most commonly used electrospray ionization (ESI), works better at lower flow rates. Therefore, analytical columns of 2.1 mm i.d. are being used which can reduce the mobile phase consumption by a factor of five or more.

The other principle of GAC listed above was the use of “safer” solvents. Mixtures of ACN/water are still the preferred extraction solvent in mycotoxin analysis. In a proficiency test about the determination of multiple mycotoxins in cereals executed in 2013 by the European Reference Laboratory (EURL) for Mycotoxins, close to 70 laboratories participated [10]. Of those, only 22 laboratories used a multi-mycotoxin method of analysis and 17 of the 22 used an ACN/water mixture for extraction. A “safer” alternative is ethyl acetate (EtOAc). It is less toxic, readily available and less expensive. Of course, to be useful as extraction solvent it needs to provide sufficient solubility for the analytes. That it is not miscible with water is not a disadvantage for extraction.

In this paper, a method of analysis is presented for the simultaneous determination of the mycotoxins Deoxynivalenol (DON), HT-2 toxin, T-2 toxin, and Zearalenone (ZON) in unprocessed cereals. Extraction is accomplished with an EtOAc/water system. To limit EtOAc consumption, a small test portion size is used. The method has been thoroughly validated in-house and through a collaborative trial and shown to perform well. The logarithm of the partition coefficient (octanol-water; log P_OW_) of the mycotoxins listed above ranges from −0.7 (DON) to 3.6 (ZON). It is safe to assume that for other mycotoxins with log P_OW_ within the stated range, the extraction will work as well. To display the flexibility of the extraction approach the results of a quick screening method for Aflatoxin B1 (AFB1) in maize based on slight modifications of this extraction approach and flow injection-mass spectrometry (FI-MS) are also presented.

## 2. Results & Discussion

### 2.1. LC-MS Multi-Mycotoxin Method

The aim of this method of analysis is to be a tool for enforcement of existing (DON, ZON) [2] and tentative (HT-2, T-2) [11] legal limits in unprocessed cereals. At the same time, it should be quick, easy to apply and be a “greener” alternative to existing methods. The results of the in-house validation and the collaborative study will be presented and discussed together below. The collaborative study data represents results from 21 laboratories which have analyzed ten coded samples (five different materials as blind duplicates). Full details of the in-house [12] and the collaborative study [13] validation are available online.

#### 2.1.1. Isotopologues as Internal Standard (ISTD)/Matrix Effects

The use of isotopologues in MS analyses is called isotope dilution mass spectrometry (IDMS) and its utility for accurate determinations of organic substances has been recognized in the late '80s of the last century [14]. IDMS is a method of analysis of very high metrological order and enables exceptional accuracies. Yet true IDMS requires addition of the isotopologue to the test material and thorough equilibration with the analyte before extraction. This is difficult to achieve and control. Moreover, it can be prohibitively expensive. Those issues preclude this approach from being used in a routine analysis context.

We used a different approach in that we added the isotopologues to only an aliquot of the sample extract. Used in such a way, the isotopologues can only be employed for correction of matrix effects which are a major concern in LC-MS. Upstream effects like extraction efficiency and/or losses during transfer or dilution steps are not covered. Yet if the extraction conditions are under good control then this mode of usage offers the benefit of cancellation of matrix effects at very reasonable expenses [9].

For determination of the matrix effects, analyte-free materials were extracted and the extracts were spiked at four different levels with the analytes and at constant levels with the isotopologue ISTD mix [15]. After determination of the ion ratios, the matrix effects (ME) were calculated with the following formula:

(1) ME=B/C,

with *B* = slope of regression fit of ion ratios of analyte added to extracts of analyte-free material, *C* = slope of regression fit of ion ratios of analyte in neat solvent. A value of 1 indicates no matrix effect, values > 1 indicate ion enhancement, values < 1, ion suppression. For the four analytes in the six tested materials, MEs between 0.81 and 1.04 were calculated. Considering the uncertainties of the modelling and the pipetting steps, none of these values were significant with the exception of the values in oat. Also, those are still within an acceptable range. This is evidence of the validity of the approach to add the isotopologues after extraction.

#### 2.1.2. Recovery/Trueness

The recovery (Rec), the ratio of observed content to expected, of this method of analysis was determined following a similar scheme as above. The six tested analyte-free materials were spiked with the four analytes at four different levels before extraction. All of this was done in duplicate, resulting in 48 samples. After sufficient equilibration (data not shown), these samples were extracted and ion ratios were determined. The method recovery was then calculated as:

(2) Rec=A/C,

with *A* = slope of regression fit of ion ratios of analyte added to analyte-free material, *C* = slope of regression fit of ion ratios of analyte in neat solvent. Recoveries between 0.79 and 1.07 were calculated. This is well within accepted ranges [16].

Of more importance is the trueness of a method of analysis. While recovery can be estimated from spiked materials with the spiked amount being the expected amount, the determination of trueness necessitates the knowledge of a “true” value. This true value can be the certified value of a Certified Reference Material (CRM) or a value determined with a reference method [17].

To this end, for the collaborative study two test materials for which reference values were determined by exact-matching double IDMS (EMD-IDMS) were included. The process of EMD-IDMS is described in detail by Breidbach et al. [18] and in the study report [13]. Table 1 displays the results of the determination of the two reference materials (RM). The bias between the study results and the assigned values is only significant (confidence interval does not include zero) for DON and HT-2 and amounts to −11% in RM I and −8% in RM II for DON, and −11% in RM II for HT-2. This demonstrates better than recovery experiments the small to negligible systematic error achievable by the combination of EtOAc as extraction solvent, isotopologues as surrogate ISTD and LC-MS for detection.

#### 2.1.3. Precision

The variability in the results of a method of analysis, its precision, can be determined under two extreme conditions: repeatability and reproducibility. Repeatability conditions describe the minimum variability inherent in such a method determined through repeated measures in a short span of time keeping other contributing factors, such as operator, instrumentation, calibration, etc., constant. Maximum variability will be recorded under reproducibility conditions when all those factors are varied during a collaborative study. The between-laboratory variability adds then onto the repeatability variability.

The repeatability standard deviations were determined in-house through analysis of three different naturally-contaminated test materials (designated as EFL1, EFL2, and EFL3 in Table 2) 20 times each. For DON, the relative repeatability standard deviations (RSD_r_) ranged from 15% at 73 µg/kg to 6% at 427 µg/kg. The ranges for HT-2 were from 11% at 27 µg/kg to 6% at 174 µg/kg; for T-2, from 16% at 7 µg/kg to 6% at 45 µg/kg; and for ZON, from 18% at 6 µg/kg to 4% at 483 µg/kg.

The results for the collaborative study are listed in Table 2. It is apparent that the RSD_r_ values are in line with the values found in-house with three exceptions: T-2 with values of 27% and 35%, and ZON with 32%. All three exceptions are connected to very low contamination levels. A similar picture unfolds for the relative reproducibility standard deviations (RSD_R_). For most of the analyte /matrix combinations, the values are at acceptable levels except for T-2 with 44% and 88%, and ZON with 98% and 65%. The two T-2 values and the ZON value of 98% are related to the same low contamination levels as mentioned above.

The second ZON value of 65% in material IRMMFEED is apparently not related to the contamination level but to the complexity of the matrix. There is a second material (EFL1) with a very similar contamination level for which the RSD_R_ is less than half at 31%. That for both materials the RSD_r_ is very similar points to a large between-laboratory variability for IRMMFEED. The higher matrix complexity of IRMMFEED was a challenge for the separation capabilities. Based on prescribed resolution requirements, the results of the laboratories could be separated into two groups: those for which the requirements were met and those for which they were not. For test material EFL1, there was no significant difference between the overall means of the two groups; for test material IRMMFEED there was. The existence of two distinct groups explains the much higher between-laboratory variability and underlines the need for proper chromatographic separation.

#### 2.1.4. Other Method Validation Parameters

In-house, the following other validation parameters were determined: for selectivity, the six materials, which were free of all analytes, were extracted as is and after spiking with the analytes at the lowest calibration level. Analyte peaks were detected in none of the chromatograms of the unspiked and in all of the spiked materials. During calibration experiments at six equidistant levels within the working ranges (in µg/kg: DON 200–2560, HT-2 25–400, T-2 15–240, ZON 50–240), performed on six different days, no deviations from linearity were found.

**Figure 1:**
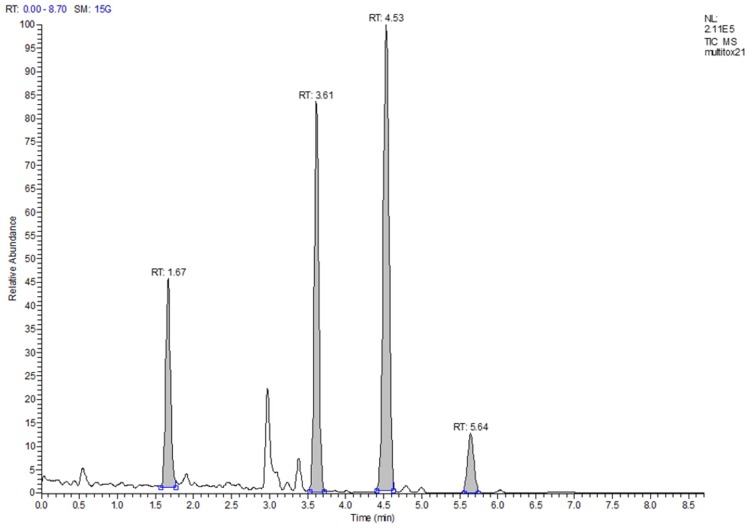
Total ion current chromatogram of a cereal mix with low contamination of DON, HT-2, T-2, and ZON; the majority of the peak signals stems from the isotopologues.

**Figure 2:**
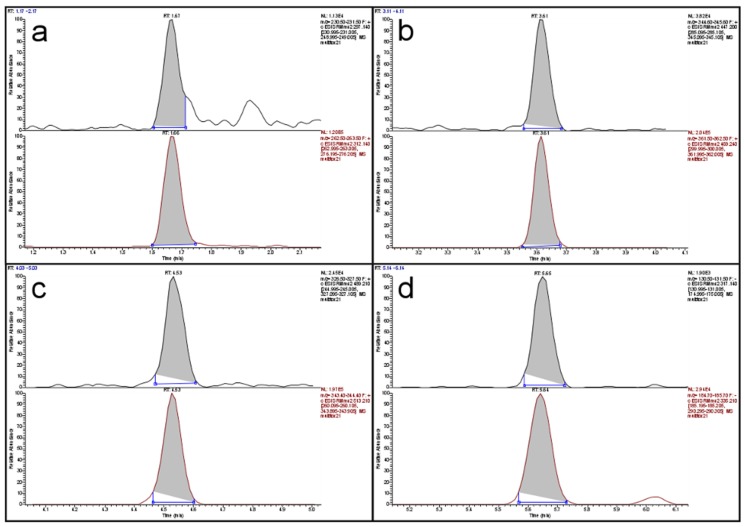
Extracted ion current chromatograms of: (**a**) DON; (**b**) HT-2; (**c**) T-2; and (**d**) ZON of a cereal mix with low contamination; in each panel the analyte (**top**) is depicted above its respective isotopologue (**bottom**).

Limits of detection (LOD) were determined from the data of the recovery experiments based on ISO 11843 Part 2 [19]. Since the estimated LODs (in µg/kg: DON < 47, HT-2 < 9, T-2 < 3, ZON < 5) were much smaller than the targeted working ranges, no more resources were invested in determining more precise LOD values. The same was the case for the determination of the limit of quantification (LOQ). These values were also estimated from the recovery data. Since almost all LOQs, with the exception of the value for DON in maize, were below the targeted working range, no more resources were invested. The LOQ for DON in maize was 340 µg/kg and almost twice as large as the next lower value (190 µg/kg in rice). The reason was an inconsistency in the recovery data for maize. Since the legislative limit for DON in unprocessed maize is 1750 µg/kg, even a “true” LOQ of 340 µg/kg would not pose a problem. Figure 1 shows a total ion current (TIC) chromatogram of the separation of a cereal mix with a fractional content of 23% maize. The peaks of the four analytes are well shaped and resolved with a total run time of 8.7 min. Figure 2 depicts the extracted ion current (EIC) chromatograms of the four analytes and their respective isotopologues of the same run as in Figure 1. The top chromatogram in panel (a) of Figure 2 shows the EIC of DON at a contamination level of about 90 µg/kg. Obviously, the signal-to-noise ratio is already very favorable at this level which indicates that the LOQ of 340 µg/kg is a very conservative estimate.

### 2.2. Quick Screening Method

To investigate the distribution of AFB1 in about 200 kg of contaminated whole grain maize, a quick screening method for 10 g samples was needed. Since the EtOAc extraction approach worked so well, it was adapted and tested in this context. To keep overall solvent consumption low, the method should work with small test portions without generating a high sub-sampling variability. Therefore, the preparation of aqueous slurries of the 10 g samples was tested.

Nine test portions equivalent to 1 g of maize each out of a slurry prepared from 10 g contaminated whole grain maize were measured with a routine LC-MS method to assess the sub-sampling variability. The relative standard deviation (RSD) of results of the nine determinations was 3% based on the peak area of AFB1. This was seen as sufficiently small to continue with slurries and test portions of 1 g maize equivalent.

#### 2.2.1. Extraction Efficiency

To speed up the extraction process, the sodium sulfate (Na_2_SO_4_), which was previously added after sonication in the LC-MS procedure, adding 10–20 min of crystallization time to the extraction, was now added before avoiding the additional wait. Whether this change influenced the extraction efficiency (EE) was determined following a similar scheme as described above. Out of a slurry of contaminated whole grain maize, four aliquots were spiked with AFB1 at two levels in duplicate before extraction and four were spiked at the same levels after extraction. The spiked and two unspiked extracts were then measured with LC-MS to obtain the peak areas of AFB1 and to calculate EE with the following formula:

(3) EE=A/B,

with *A* = slope of regression fit of peak areas of AFB1 added before extraction and *B* = slope of regression fit of peak areas of AFB1 added after extraction. An extraction efficiency of 89% ± 5% (95% confidence) was calculated, which is in line with findings of Mol et al. [20] and shows that the early addition of the Na_2_SO_4_ has no significant negative effect.

#### 2.2.2. Flow Injection-MS

While chromatographic separation before MS detection has many advantages and is strongly recommended for quantitative assessments, it is time consuming. One of those advantages is certainly the reduction of matrix effects because fewer compounds will coelute with the analyte. However, for a qualitative screening method, this can be sacrificed to speed up analysis even more.

**Figure 3:**
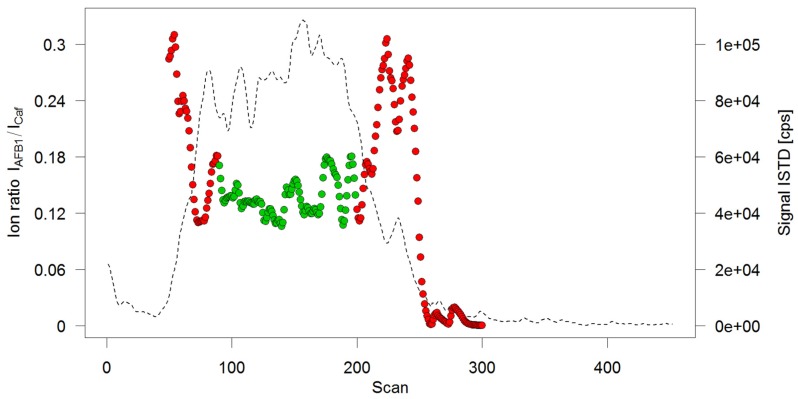
Ion ratio profile of a flow injection analysis. Solid circles depict the calculated ion ratios per scan, only the green ones have been retained for averaging. Superimposed is the chronogram of the internal standard (ISTD) caffeine (broken line).

Therefore, the test solutions were directly delivered to the MS by injecting them into the spray solvent flow. Since ionization efficiencies of electrospray improve at lower flow rates, and to save solvent, a flow rate of 20 µL/min was chosen. This led to broad, irregular elution profiles (see Figure 3) which could not be integrated automatically. To obtain a concentration-dependent response, a different approach was chosen: Scan-based ion ratios, which had shown to lead to exceptional accuracies in IDMS measurements [21], were utilized to automatically evaluate the flow injection measurements. For this, the ion ratio of the AFB1 signal over an ISTD signal (Caffeine was chosen as ISTD) was calculated as follows: for each scan event measuring the transition m/z 313->241 (AFB1) the signals of the preceding and the successive scan event measuring the transition m/z 195->138 (Caffeine) were averaged. The signal of m/z 313->241 was then divided by the average of m/z 195->138 to obtain the ion ratio for the respective scan. When plotting the signal of the ISTD, a broad peak with flat top could be observed (Figure 3). Averaging all ion ratios belonging to that flat-top region then provides a mean ion ratio which is a representation of the amount-dependent analyte response.

#### 2.2.3. AFB1 Distribution and Standard Addition

**Figure 4:**
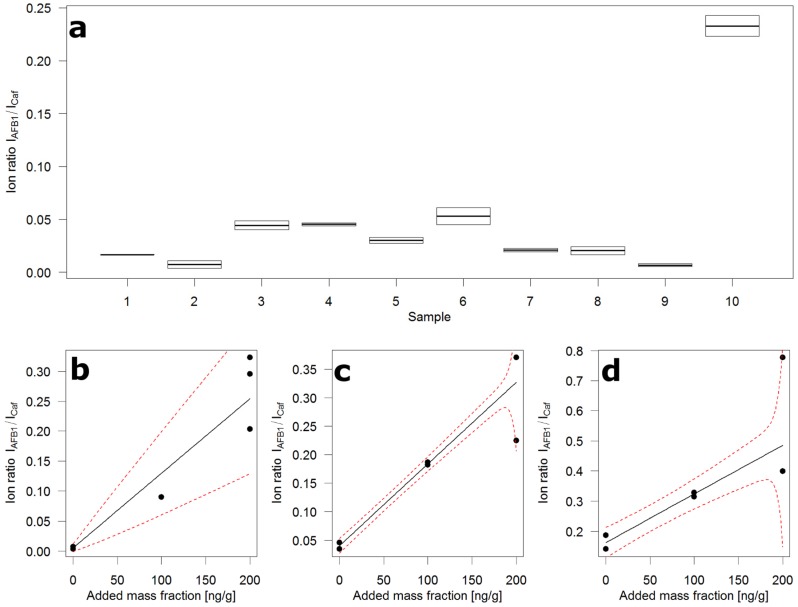
Ion ratio distribution over ten 10 g samples of contaminated crushed maize and standard addition plots. (**a**) Box plot of duplicate flow injections of ten random sub-samples, boxes extend to the two measured ion ratios, horizontal line depicts the mean of the two ion ratios; (**b**) Standard addition plot of sample 2 in (**a**); (**c**) Standard addition plot of sample 6 in (**a**); (**d**) Standard addition plot of sample 10 in (**a**); black circles—ion ratio (mean of two injections), black line—regression fit, red broken line—prediction interval.

Panel (a) of Figure 4 shows the distribution of AFB1 over ten 10 g samples drawn at random from 10 kg of crushed maize and submitted to the flow injection analysis. The 10 kg were sampled from the 200 kg of contaminated whole grain maize according to the incremental sampling scheme (100 × 100 g) described in the corresponding European Union (EU) regulation [16] for large lots. Next to samples containing very little AFB1, there is one sample containing very much. This is evidence of the known heterogeneity of AFB1 contamination. To obtain an idea of the contamination levels, the slurries of samples 2, 6, and 10 were submitted to standard addition (STDADD) analysis.

The use of STDADD became necessary because of the uncontrolled and severe matrix effects that come along with the flow injection measurement. The additional effort was limited because several aliquots were available for extraction from each slurry. For STDADD experiments, six aliquots, representing 1 g of maize each, were used. Two were extracted in their native state, the other four were spiked with AFB1 at two levels in duplicate. For sample 2 (Panel (b), Figure 4), one medium and three high-level spikes were produced due to a pipetting error.

The calculated mass fractions of AFB1 in the three samples were: 4 (0–76) µg/kg in sample 2; 28 (16–40) µg/kg in sample 6; and 100 (59–165) µg/kg in sample 10. The values in parentheses represent the ~95% confidence interval around the calculated value. These intervals have been determined from the computed prediction intervals of the STDADD experiments. The confidence intervals around the predicted value are unsymmetrical by definition. The value zero in the interval of sample 2 shows that the calculated value is below the detection capabilities of the analytical method. The width of the intervals indicates the semi-quantitative nature of this approach. Yet it appears to be valuable as a tool to screen large numbers of samples. In this study, the focus was on AFB1 which is notoriously heterogeneously distributed and has low legal limits. This approach could easily be adapted to other mycotoxins, for instance fumonisins, and certainly DON, HT-2, T-2, and ZON. The use of 1% trifluoroacetic acid in the slurries boosts extraction efficiencies of fumonisins and prevents, due to the low pH, enzymatic activities which appear to convert T-2 toxin through deacetylation to HT-2 toxin.

## 3. Conclusions

In conclusion, it can be said that the presented approach is:

- greener than many existing approaches because it replaces ACN through EtOAc, minimizes the consumption of organic solvent, and produces less chemical waste;
- simple and quick because a time-consuming clean-up step is avoided and sample processing is easily scaled up or down;
- adaptable to other applications without too much effort;
- showing favorable performance characteristics and is fit-for-purpose as a tool to enforce EU mycotoxin legislation.

The fitness-for-purpose is also evidenced through the fact that the presented LC-MS method of analysis has been published by the European Committee for Standardization (CEN) as European standard method of analysis [22].

## 4. Materials and Methods

### 4.1. Chemicals and Materials

All chemicals were purchased from either Sigma-Aldrich (Overijse, Belgium) or VWR (Leuven, Belgium) and were of at least analytical grade. For mobile phases LC-MS CHROMASOLV grade water and methanol (MeOH), formic acid (FA), ammonium formate (NH_4_FA) (all LC-MS grade, Fluka, Sigma-Aldrich), and LC-MS grade acetonitrile (ACN; VWR) were used. Deionised water was generated by a MilliQ system (Millipore, Overijse, Belgium). All tested materials came from the material pool of the EURL for Mycotoxins of the Joint Research Centre (JRC) of the European Commission (EC).

The mycotoxins Deoxynivalenol (DON), HT-2 toxin, T-2 toxin, Zearalenone (ZON), Aflatoxin B1 (AFB1), and the isotopologues ^13^C_15_-DON, ^13^C_22_-HT-2, ^13^C_24_-T-2, and ^13^C_18_-ZON were purchased from Biopure (Romer Labs, Getzersdorf, Austria) as ready-to-use solutions and were stored at the recommended temperatures.

From those solutions, a mixed stock solution of 3.2 µg/mL DON, 0.5 µg/mL HT-2 toxin, 0.3 µg/mL T-2 toxin, and 0.3 µg/mL ZON in acetonitrile was prepared and stored. This stock solution was freshly diluted for every calibration task. A mixed ISTD solution with the same concentrations of the respective ^13^C-isotopologues in acetonitrile was also prepared and used undiluted. These two solutions were stable for at least three months in the dark between 2 °C and 8 °C.

For buffered mobile phases, an equimolar mix of NH_4_FA and FA (pH 3.7), adjusted to 6 mol/L in water, was added to the solvents.

### 4.2. Sample Preparation for LC-MS

In a 50 mL conical screw-cap polyethylene centrifuge tube (VWR), 2 g of test material (comminuted to <500 µm particle size) were fully suspended in 8 mL of water. Then 16 mL ethyl acetate (EtOAc) was added and after a brief, hard shake, the mixture was sonicated for 30 min. After sonication, 8 g of Na_2_SO_4_ were added. The mixture was again shaken hard and then left for 10 to 20 min to allow the Na_2_SO_4_ to crystallize. To settle particulate matter and aid phase separation, the tube was centrifuged at a relative centrifugal force (RCF) of 3200 g for at least 1 min. 500 µL of the clear supernatant were transferred to a silylated auto sampler vial (2 mL, Supelco, Sigma-Aldrich), 25 µL of ISTD mix were added and the content of the vial was evaporated to dryness with a stream of dry nitrogen (boil-off) at 60 °C. The dry residue was reconstituted with 250 µL MeOH/FA (999/1, *v*/*v*) and 250 µL water/FA (999/1, *v*/*v*), in that order. Initial reconstitution with the pure organic mobile phase significantly improved the dissolution of the more hydrophobic analytes. Turbidity of the injection solutions, often seen in these reconstituted extracts, did not negatively affect column lifetime in our experience.

### 4.3. Sample Preparation for Quick Screening

Ten g of maize (whole grain or comminuted) were high-speed blended (Ultra Turrax, IKA-Werke, Germany) with 20 g water/trifluoroacetic acid (TFA) (99/1, *v*/*v*) to obtain a smooth, semi-viscous slurry; 3 g of that slurry (representing 1 g of test material) were transferred to a 50 mL conical screw-cap polyethylene centrifuge tube, and 8 mL ethyl acetate (EtOAc) and 4 g Na_2_SO_4_ were added. The tube was capped, shaken hard, and then sonicated for 30 min. Right after, the tube was centrifuged at RCF of 3200 g for 2 min. Five hundred µL of supernatant, together with 25 µL of a 400 µg/mL solution of caffeine, used as ISTD, were dried down in a silylated auto sampler vial with nitrogen as described above. The dry extract was reconstituted with 250 µL ACN/water/NH_4_FA pH 3.7 (90/9/1, *v*/*v*/*v*) and 250 µL water/NH_4_FA pH 3.7 (99/1, *v*/*v*/*v*).

To determine contamination levels, known amounts of AFB1 were added to aliquots of the slurry before extraction. After allowing for 60 min of equilibration between slurry and added analyte, these spiked standard addition samples were extracted as described above.

### 4.4. LC-MS Measurements

The LC consisted of two LC-20 AD pumps (Shimadzu, Belgium) for a high-pressure binary gradient and an Accela Auto Liquid Sampler (ALS) (Thermo Scientific, Belgium). The gradient delay volume was minimized through the use of an ASI binary static micro-mixer with a 25 µL cartridge (Supelco, Sigma-Aldrich) and 0.13 mm ID tubing for all fluid connections. The ALS was equipped with a 5 µL sampling loop. As analytical column, an Ascentis Express C18 (75 × 2.1 mm, 2.7 μm particle size; Supelco, Sigma-Aldrich) was employed. Mobile phase A was water/FA (999/1, *v*/*v*) and mobile phase B MeOH/FA (999/1, *v*/*v*). The gradient conditions were as follows: 0 min 8% B, 2 min 57% B, 6 min 61% B, 6.1 min 95% B, 7.6 min 95% B, 7.7 min 8% B, 8.7 min 8% B at a flow rate of 0.3 mL/min. The column was maintained at 40 °C during analysis. This gradient was designed with optimal resolution and shortest analysis time for just the four mycotoxins in mind and was obtained using computer-aided design [23].

A TSQ Quantum Ultra triple-quadrupole mass spectrometer with IonMax HESI2 interface (Thermo Scientific, Erembodegem-Aalst, Belgium) was used in SRM mode with the settings listed in Table 3. The MS was calibrated according to manufacturer’s recommendations and source/analyser settings have been optimized using “Design of Experiments” with the full LC-MS setup. Other source settings were: vaporizer temp. 350 °C, capillary temp. 320 °C, sheath gas press. 30 arbitrary units (a.u.), auxiliary gas press. 10 a.u., sweep gas press. 10 a.u. The collision gas was argon at 1.5 mTorr. The data acquisition was segmented to limit the number of acquired transitions and enable longer dwell times per analyte while maintaining a fast-enough scan rate to obtain 15–20 scans per peak.

### 4.5. Flow Injection Measurements

To perform the flow injection measurements, a LC-20 AD pump equipped with a low-pressure gradient unit and the standard mixer (Shimadzu Benelux, 's-Hertogenbosch, The Netherlands) delivered the mobile phase at a flow rate of 20 µL/min. The mobile phase consisted of ACN/water/NH_4_FA pH 3.7 (45/54/1, *v*/*v*/*v*). A HTC PAL (CTC Analytics, Switzerland) with a 10 µL loop was used to inject test solutions into the solvent flow. The loop was overfilled three times. The same TSQ Quantum Ultra triple-quadrupole mass spectrometer with IonMax HESI2 interface (Thermo Scientific, Erembodegem-Aalst, Belgium) was used in SRM mode. The source settings were as follows: vaporizer temp. off, capillary temp. 300 °C, sheath gas press. 10 a.u., auxiliary gas press. 1 a.u., sweep gas press. 1 a.u. The collision gas was argon at 1.5 mTorr. The spray voltage was set to 3 kV and the tube lens to 110 V. The following transitions (respective collision energies in parentheses) were continuously measured for the run time of 2 min: for Caffeine m/z 195->83 (30 eV), 195->110 (30), 195->138 (30); for AFB1 m/z 313->241 (37 eV), 331->270 (29).

### 4.6. Computations

All statistical computations were performed using the free software “R” [24]. For all calculations involving responses, either ion ratios of peak area of analyte over peak area of respective isotopologue (LC-MS) or ion ratios of ion intensity of analyte over ion intensity of ISTD (flow injection) were used. Due to the heteroscedasticity, regression fits of those ion ratios over amount of analyte were performed using generalized least-squares modelling with variance function class “varConstPower” [25]. From the modelled variance function, prediction intervals were computed which allowed assigning proper confidence intervals around predicted amounts of analyte. The computations for LOD/LOQ and prediction intervals were based on ideas in an S-PLUS script published by O’Connell [26]. For the evaluation of the flow injection measurements, the MS data was converted to mzML [27] format, which was then further processed with a “R” script. All “R” packages and scripts used for this study are available from either [24] or on request from the author.

### 4.7. Method Validation

The LC-MS method underwent first a thorough single-laboratory validation. In accordance to Regulation (EC) No. 882/2004 [28], selectivity, linearity, matrix effects, working range, LOD, LOQ, repeatability, intermediate precision, recovery, trueness, and robustness were investigated. This was done in the following materials: maize, wheat, oat, rice, soy and a cereal-based compound feed. For matrix effect and method recovery determination, different amounts of the analytes and equal amounts of ISTD were either spiked into materials free of the analytes before extraction (Set A) or after extraction of the analyte-free materials (Set B). After regression analysis, the slopes of the signals of the sets A and B were then compared with the slopes of a calibration done in neat solvent (Set C). Comparing slope A and C indicated method recovery, while comparison of B and C determined the extent of matrix effects [15].

The data of these spiking experiments were also used to compute LOD based on ISO 11843 Part 2 [19]. LOQs were computed based on an extension of the concept of ISO 11843 towards variance function estimation [25]. For repeatability and intermediate precision, naturally contaminated cereal mixes were prepared and measured 20 times on the same day (repeatability) and once each on a total of eight days by three different operators (intermediate precision). A detailed validation report is available online [12]. The method was then further validated through a collaborative trial [13]. This method and the results of the collaborative trial have recently been published as a European Standard by the European Committee for Standardization (CEN).

The sole purpose of the flow injection method was to screen maize samples for the presence of AFB1. Its validation was therefore very limited and included only the determination of the extraction efficiency in maize.

## Figures and Tables

**Table 1:** The assigned reference values from EMD-IDMS and the collaborative study results.

Analyte	Assigned Value		Study Result					
	*xa* (µg/kg)	u(*xa*) ^1^ (µg/kg)	Overall Mean (µg/kg)	*sR* ^2^ (µg/kg)	(µg/kg) ^3^	*A* ^4^	-*A sR* ^5^ (µg/kg)	+*A sR* ^6^ (µg/kg)
**Reference Material I (EFL2)**								
DON	282	13	250	33	−32	0.47	−47	−16
HT-2	51	3	49	12	−2	0.48	−8	4
T-2	18	1	18	4	0	0.47	−2	2
ZON	28	2	30	6	2	0.46	−1	5
**Reference Material II (EFL3)**								
DON	605	24	559	67	−46	0.46	−77	−15
HT-2	201	7	178	23	−23	0.45	−34	−13
T-2	52	2	50	6	−2	0.46	−5	1
ZON	445	8	430	49	−15	0.46	−38	7

^1^ combined standard uncertainty of the assigned value; ^2^ reproducibility standard deviation; ^3^ bias (overall mean—assigned value); ^4^ factor for ca. 95% confidence interval; ^5^ lower limit 95% confidence interval around bias; ^6^ upper limit 95% confidence interval around bias.

**Table 2:** Results of the collaborative study.

Material ^1^	Overall Mean ^2^ (µg/kg)	s_r_^3^ (µg/kg)	RSD_r_ (%)	s_R_^4^ (µg/kg)	RSD_R_ (%)
DON					
EFL1	88.5	9.5	11	17.0	19
EFL2	250.0	13.6	6	33.3	13
EFL3	558.6	30.1	5	66.9	12
IRMMCER	135.8	8.2	6	23.0	17
IRMMFEED	281.8	19.9	7	33.1	12
HT-2					
EFL1	38.0	3.4	9	6.2	16
EFL2	49.1	3.4	7	12.0	25
EFL3	177.6	13.5	8	23.2	13
IRMMCER	53.1	8.1	15	12.4	24
IRMMFEED	22.0	3.3	15	6.3	29
T-2					
EFL1	12.1	1.7	14	3.9	32
EFL2	17.7	1.6	9	4.4	25
EFL3	50.3	3.1	6	6.5	13
IRMMCER	7.0	1.8	27	3.1	44
IRMMFEED	3.5	1.2	35	3.1	88
ZON					
EFL1	13.9	2.0	15	4.3	31
EFL2	30.5	2.9	10	6.0	20
EFL3	430.0	25.0	6	49.3	12
IRMMCER	3.4	1.1	32	3.3	98
IRMMFEED	15.9	1.7	11	10.4	65

^1^ more information about the materials in [13]; ^2^ average of all retained lab results; ^3^ repeatability standard deviation; ^4^ reproducibility standard deviation.

**Table 3:** Mass spectrometry (MS) source and analyser settings.

Item	Segment 1	Segment 2	Segment 3	Segment 4
Run time (min)	0–2.6	2.6–4.1	4.1–4.9	4.9–8.7
Analyte	DON + ^13^C_15_-DON	HT-2 + ^13^C_22_-HT-2	T-2 + ^13^C_24_-T-2	ZON + ^13^C_18_-ZON
Adduct	Protonated	Sodium	Sodium	Deprotonated
Transitions (m/z)(Collision Energy [eV])	297->231 (16),297->249 (13),312->263 (9),312->276 (9)	447->285 (22),447->345 (20),469->300 (19),469->362 (18)	489->245 (30),489->327 (25),513->260 (26),513->344 (23)	317->131 (25),317->175 (22),335->185 (26),335->290 (21)
Dwell time [ms]	130	130	180	180
Tube Lens [V]	80	110	140	80
Polarity	Pos	Pos	Pos	Neg
Spray Voltage [V]	2800	2800	2800	2200
